# Supplementary material for: Wilm’s tumor 1 promotes memory flexibility
Source: Nat Commun. 2019 Aug 21;10:3756. doi: 10.1038/s41467-019-11781-x (PMC6704057; doi:10.1038/s41467-019-11781-x)
Supplement: Supplementary file 1 — Supplementary Information [file 41467_2019_11781_MOESM1_ESM.pdf]

Wilm’s tumor 1 promotes memory flexibility

Supplementary Information

Mariottini *et al.* 2019

Transcription Factor	Stimulated 30 min
RARA	↑
TBP	↑
EGR1 (Zif268)	↑
USF1	↑
AHR, ARNT	↑
REL	↑
TCF7	↑
APP	↑
GATA1, GATA2	↑
GATA2	↑
GFI1	↑
FOXI1	↑
HIF1A	↑
STAT1, STAT2	↑
USF1, AP1	↑
SP1, AP1	↑
NFYA	↑
PARP1	↑
PAX4	↑
PAX6	↑
PAX8	↑
SP1, USF1, AP1	↑
NR1I2	↑
USF1, SP1	↑
CTCF	↑
KLF1	↑
MIZF	↑
HMGA1	↑
HNF1A, TCF1	↑
HOXD8, HOXD9, HOXD10	↑
IKZF1	↑
MTF1	↑
RUNX2	↑
WT1	↑
TEAD2	↑
HIVEP1	↑
MYB	↑
PAX1	↑
PAX2	↑
SURF2	↑
Thymus BP	↑
XBP1	↑
YBX1	↑

Supplementary Figure 1: **Identification of WT1 as regulator of synaptic plasticity.** Summary table of transcription factors (TFs) activated 30 min after LTP induction. Each represented transcription factor on the table was activated in at least two separate biological replicates and with a fold change ≥ 1.3.

Supplementary Figure 1

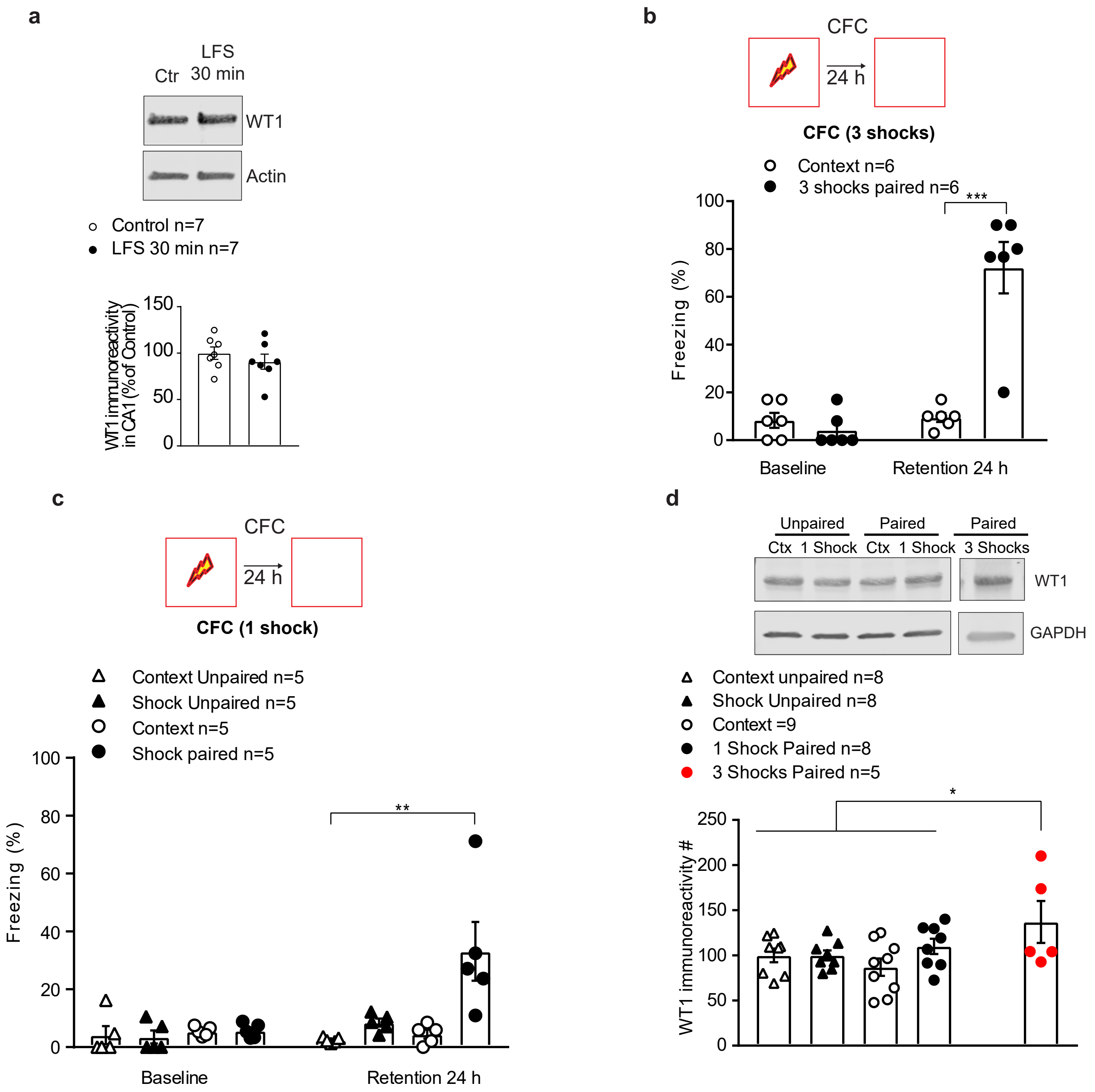

Supplementary Figure 2: Lack of change in WT1 protein levels after LFS and CFC (1 shock; Paired versus Unpaired protocol). a, Expression of WT1 did not increase in rat CA1 30 min after stimulation with LFS (paired t test: p=0.4003). **b,** Bar graph showing significant increase in freezing time for rats trained in CFC (3 shocks paired protocol) compared to control rats (Context) and tested 24 h after training (unpaired t test ***p=0.0002). These behavioral data refer to western blot in Figure 1f. **c**, Bar graph showing significant increase in freezing time for rats trained in the CFC paired versus unpaired protocol (one-way ANOVA F(7.472), **p=0.0024). **d**, Expression of WT1 did not increase in rat dorsal hippocampus 30 min after training in CFC paired versus unpaired protocol (1 shock protocol; mean ± s.e.m.). WT1 expression in rats trained in the 3 shocks CFC paired protocol was significantly different (one-way ANOVA, F(2.698), *p=0.0475). These data refer to behavioral data reported in Supplementary Figure 2c. # Shock paired groups were normalized to shock unpaired group whereas context group was normalized to context unpaired group. Data are expressed as mean ± s.e.m.

Supplementary Figure 2

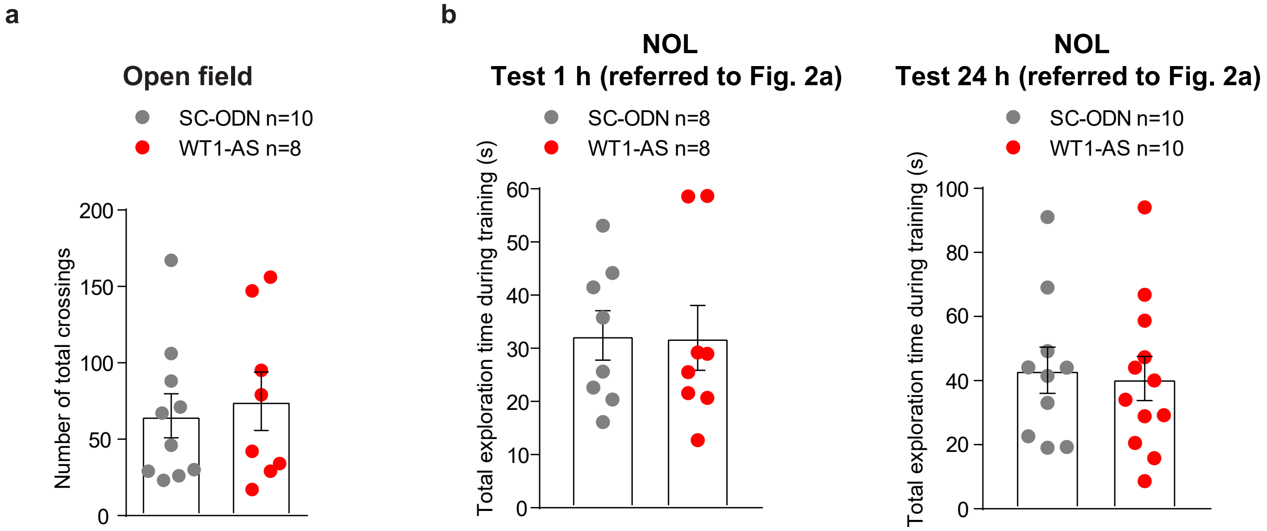

Supplementary Figure 3: Manipulation in WT1's expression does not alter rat locomotor activity and exploration during training. a, Injection of WT1-AS did not affect locomotion in an open field arena (unpaired t test, $p>0.05$). **b**, Both WT1-AS and SC-ODN- injected groups showed no difference in the total exploration time during training in a NOL task. The two different graphs refer to the two different time points (1 h and 24 h respectively) reported in Figure 2a (unpaired t test, $p>0.05$). Data are expressed as mean ± s.e.m.

Supplementary Figure 3

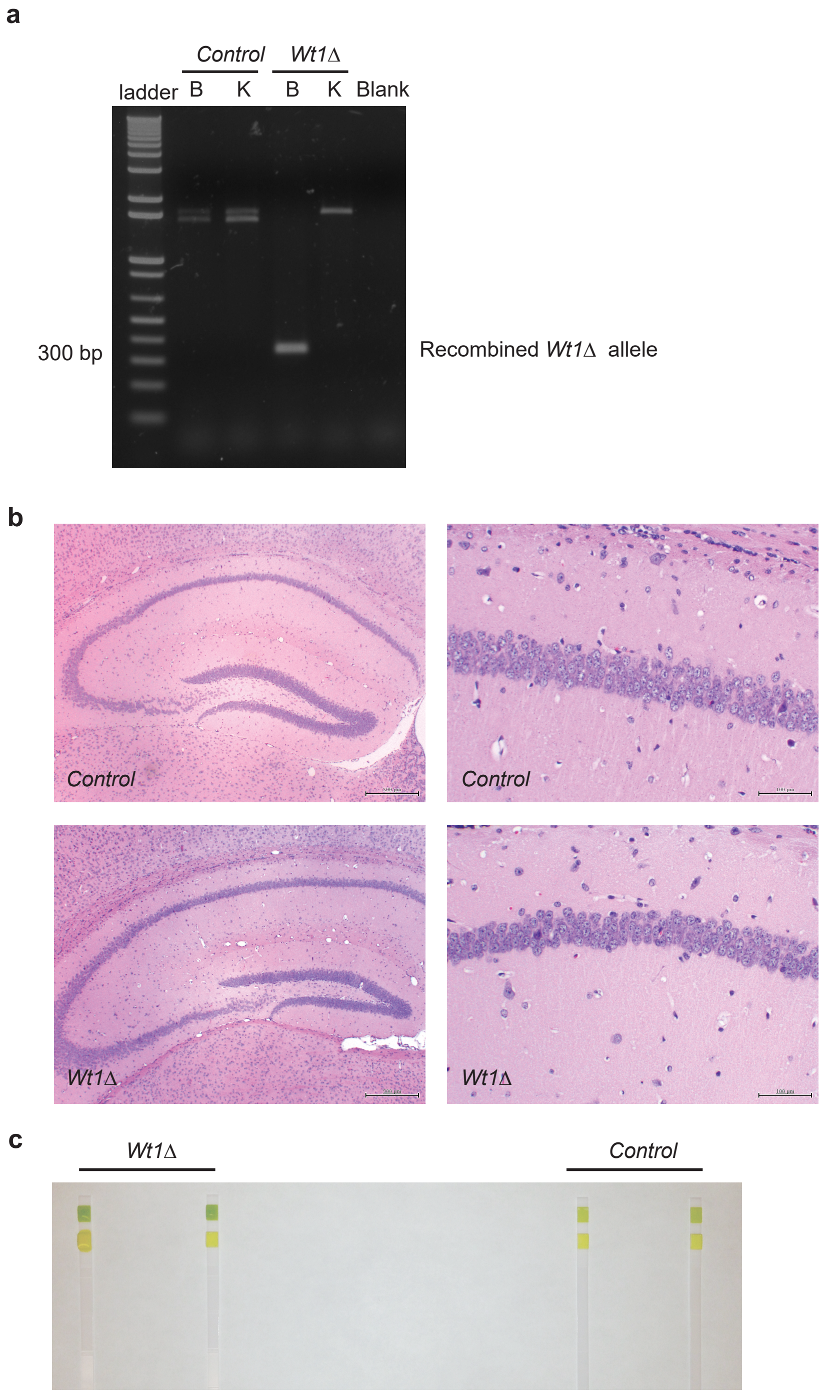

d

Genotype	ALP	ALT	AST	Creatinine	LDH	Amylase	Lipase	Albumin	Tot. protein	BUN	Uric acid	BUN/Creat	Chloride	Potassium	Sodium	Triglyceride
Control #1	169	55	89	0.3	638	1109	73	2.6	4.7	29	3	96.7	111	5.1	152	115
Control #2	160	27	80	0.3	578	1059	72	2.6	4.8	26	4.4	86.7	118	6	157	84
Control #3	99	35	118	0.3	463	1503	441	3.3	5.9	18	4.9	60	109	7.8	155	121
Control #4	99	38	46	0.3	237	952	79	3.4	6.1	19	10.5	63.3	106	>10	155	128
Wt1Δ #1	147	32	120	< 0.3	663	943	60	< 3	5.5	31	3.2	* DNR	* DNR	* DNR	* DNR	117
Wt1Δ #2	66	50	111	0.3	551	817	67	3.5	6.3	26	9.4	86.7	117	>10	167	212
Wt1Δ #3	59	29	79	0.2	677	853	61	3.1	5.5	22	6.9	110	109	9.1	154	212

Supplementary Figure 4

Supplementary Figure 4: Characterization of *Wt1*Δ mice. **a**, Expression of the recombined *Wt1*^{fl} allele (*Wt1Δ*) in the brain (indicated as “B”) and kidney (indicated as “K”) of mice as shown by PCR. Kidney samples from both groups were negative; as expected, the recombined allele was not expressed in either tissue obtained from control mice. **b**, H&E staining of adult mice hippocampal sections showed no apparent morphological differences between wild-type and *Wt1*Δ mice. **c**, Urinalysis indicated that kidney function was intact in *Wt1*Δ mice as shown by comparison of proteinuria levels with control littermates. **d**, Blood chemistry panel of control and *Wt1*Δ mice. Legend: *DNR, Did Not Report; ALP, Alkaline Phosphatase; ALT, Alanine Aminotransferase; AST, Aspartate Transaminase; LDH, Lactate Dehydrogenase; BUN, Blood Urea Nitrogen; BUN/Creat, Blood Urea Nitrogen over Creatinine Ratio.

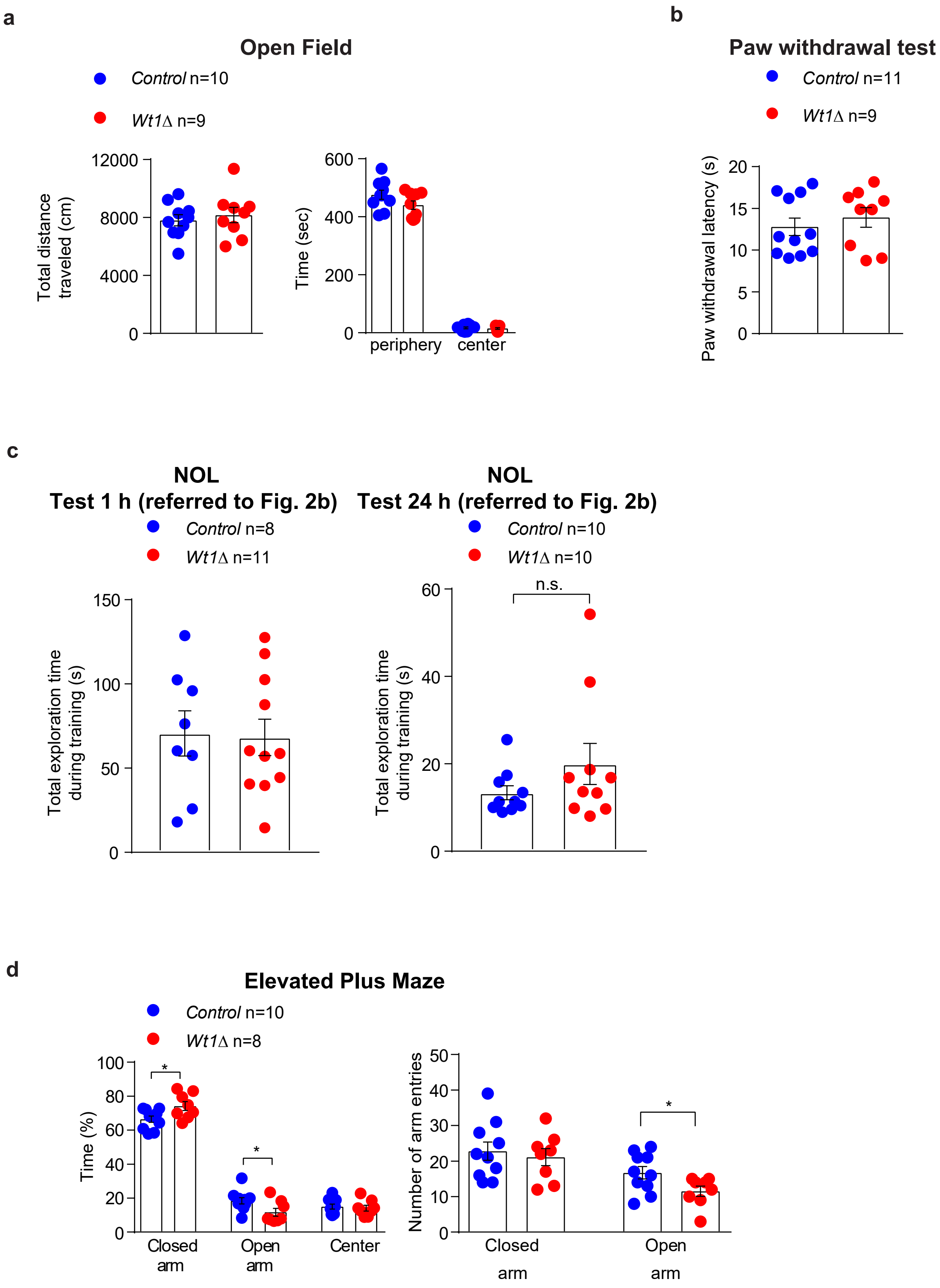

Supplementary Figure 5

Supplementary Figure 5: *Wt1*Δ mice phenotype. a, When tested in an open field, *Wt1*Δ mice showed locomotion similar to their control littermates, expressed as total distance traveled (left panel) or time spent in the periphery and center of the open field arena (right panel: unpaired t test, $p>0.05$). **b**, A paw withdrawal test was used to show that there was no difference in the nociception of *Wt1*Δ mice compared to control littermates (unpaired t test, $p>0.05$). **c**, Control and *Wt1*Δ mice showed similar total time of exploration during training in a NOL task. The two graphs refer to the two different time points (1 h and 24 h respectively) reported in Figure 2b (unpaired t test, $p>0.05$). **d**, Left panel: *Wt1*Δ mice spent significantly more time in the closed arm (left panel: unpaired t test: *p=0.0249) and correspondently significantly less time in the open arm of the elevated plus maze compared to wild-type littermates (unpaired t test: *p=0.0339). Also, *Wt1*Δ mice entered the open arm of the maze a significant lower number of times (right panel: unpaired t test, *p=0.0412). Data are expressed as mean ± s.e.m.

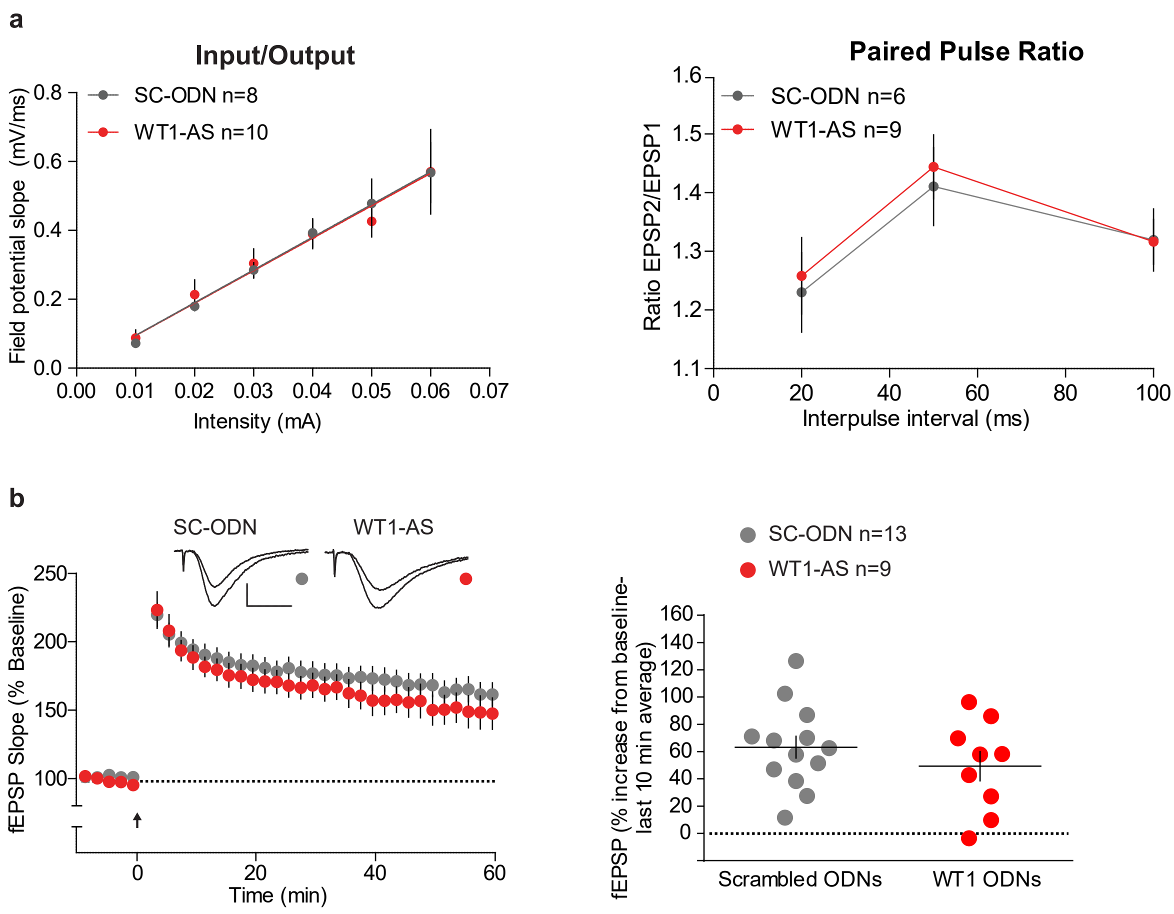

Supplementary Figure 6: WT1 knock-down rats show basal synaptic transmission and Strong-HSF induced LTP similar to control rats. a, Acute WT1 knock-down did not affect basal synaptic transmission measured through the analysis of the input/output relationship (left panel: linear regression t-test, $p>0.05$) or the paired-pulse ratio (right panel: two-way ANOVA RM, $p>0.05$) at Schaffer collateral-CA1 inputs. **b**, Injection of WT1-AS did not affect LTP induced by Strong-HFS. Representative fEPSPs show superimposed traces recorded during baseline and 60 min post-HFS. Calibrations: 0.5 mV/10 ms. The arrow indicates time of Strong-HFS delivery (left panel). Summary graph for the final 10 minutes of the recording (right panel: two-way ANOVA RM: $p>0.05$). Data are expressed as mean ± s.e.m.

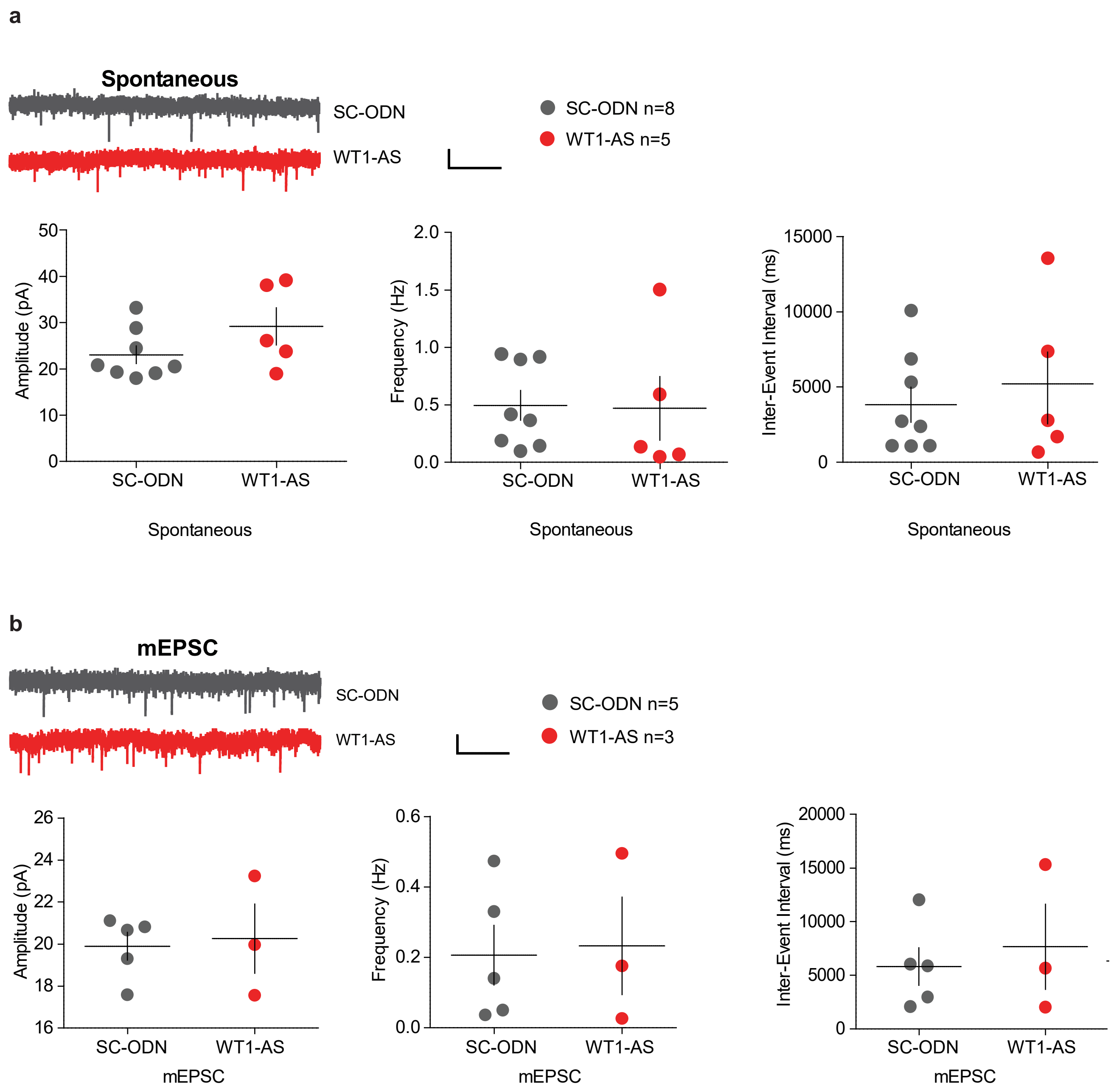

Supplementary Figure 7: WT1 knockdown does not alter spontaneous postsynaptic currents or mEPSCs in rats. a, In whole-cell recordings from area CA1 pyramidal neurons of acute slices, ODN-mediated depletion of WT1 (WT1-AS) did not affect amplitude, frequency, or inter-event interval for spontaneous currents (unpaired t tests, all p's >0.05). Calibration: 20 pA/5s. **b**, Amplitude, frequency, and inter-event interval for mEPSCs were not affected by WT1-AS treatment (unpaired t tests, all p's >0.05). Calibration: 20 pA/5s. Data are expressed as mean ± s.e.m.

a

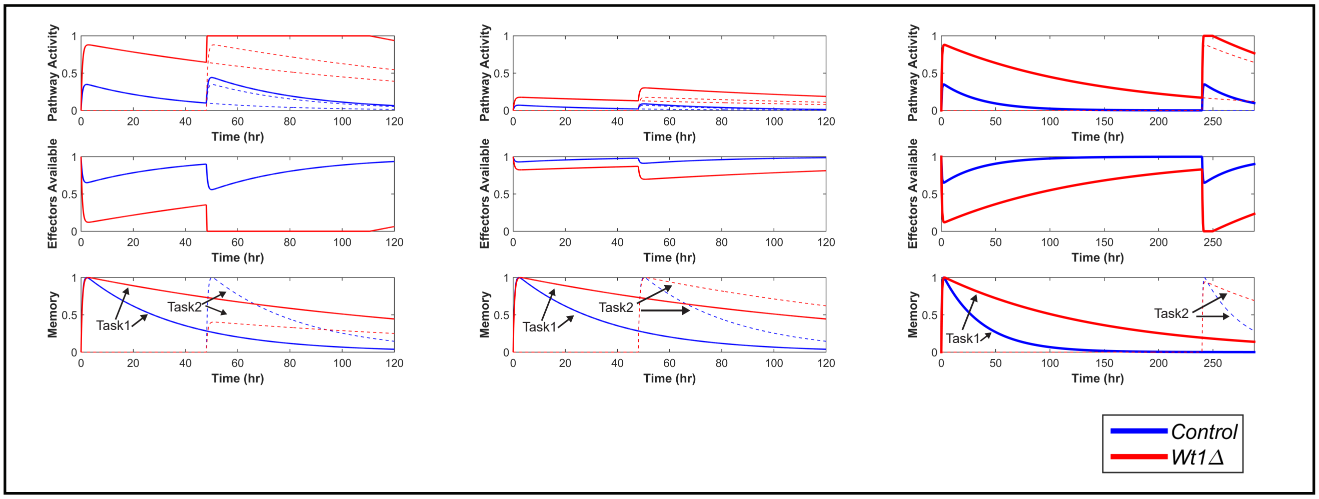

b

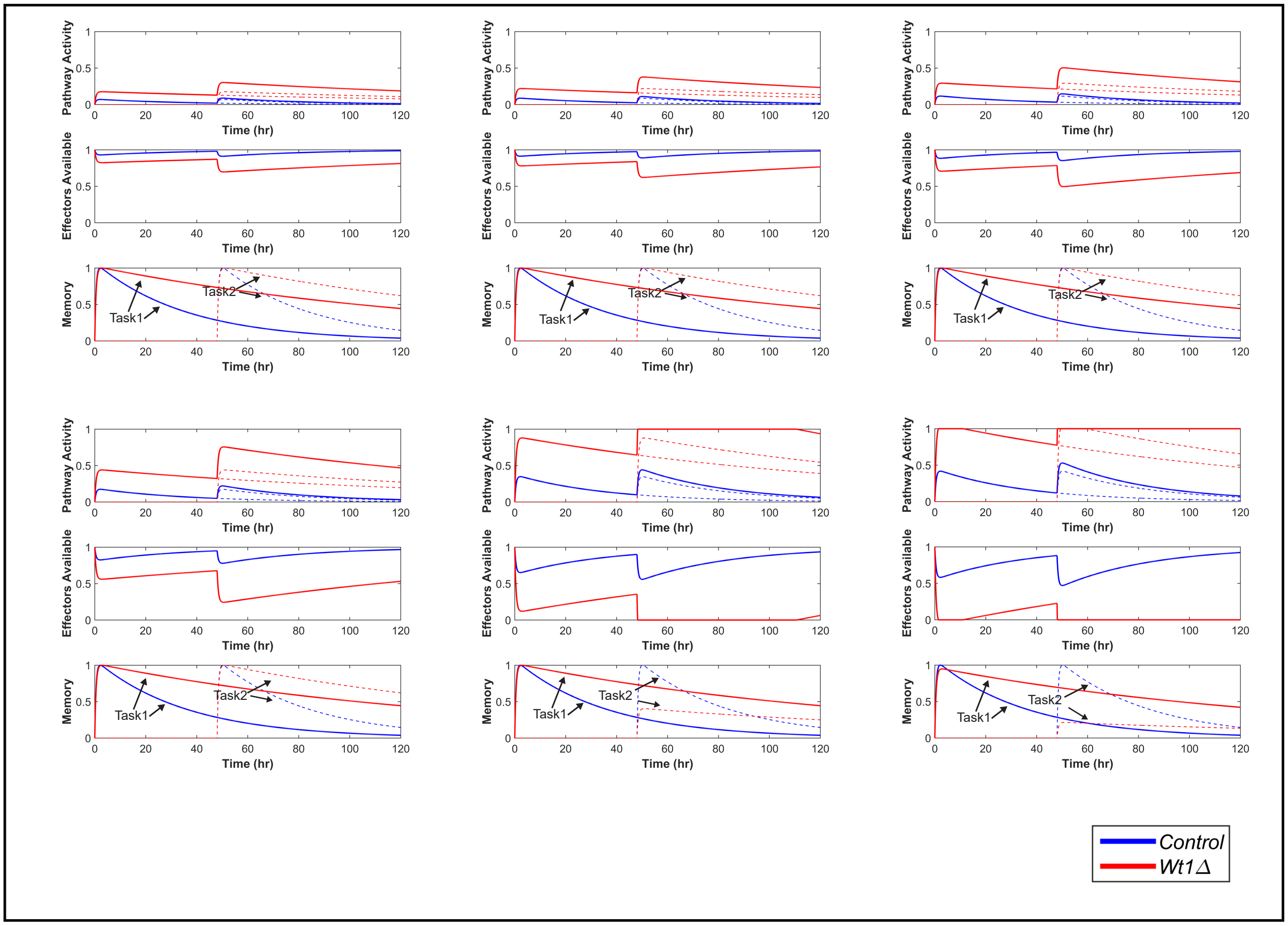

Supplementary Figure 8

Supplementary Figure 8: Control theory model of WT1-mediated effect on memory flexibility. a, Simulations using the toy control theory model. Parameter values are given in Table 2. In all panels, a first event (Task1) is simulated at t=0. The strength of the input u is taken as 0.3 in the left column and the right column of panels, and as 0.05 in the middle column of panels. The second event (Task2) is simulated at 48 h in the left and middle column of panels, and 10 days in the right column of panels. In Pathway Activity plots, the skinny dotted lines refer to contributions for individual events, and the thick solid line is the total from all events. In Memory plots, thick solid lines denote Task 1, whereas thick dashed lines denote Task 2. **b**, Effects of parameter sweeps: here the value of the input magnitude *u* was varied from 0.05 to 0.3, in increments of 0.05. As *u* is increased, the effect of saturation upon a 2nd stimulation becomes evident.

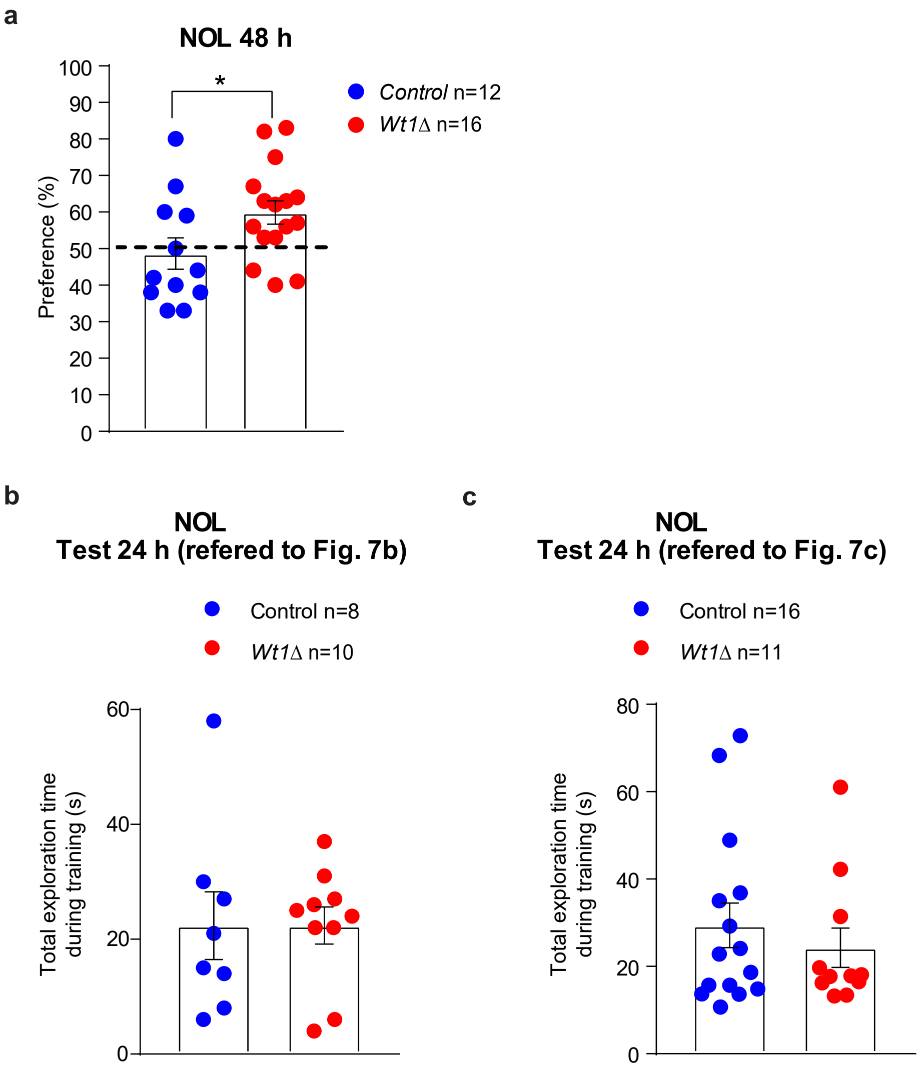

Supplementary Figure 9: *Wt1*Δ mice show enhanced LTM for NOL at 48 h. a, *Wt1*Δ mice, when compared to their wild type littermates, are significantly different for their preference for the new location when tested 48 h after training (unpaired t test, * p=0.0410. Dashed line indicates 50% preference). **b**, Both *Control* and *Wt1*Δ mice showed no difference in the total time of exploration during training in a NOL task during a sequential learning protocol (referred to Figure 7b; unpaired t test, p>0.05). **c**, Both *Control* and *Wt1*Δ mice showed no difference in the total time of exploration during training in a NOL task during a sequential learning protocol (referred to Figure 7c; unpaired t test, p>0.05). Data are expressed as mean ± s.e.m.

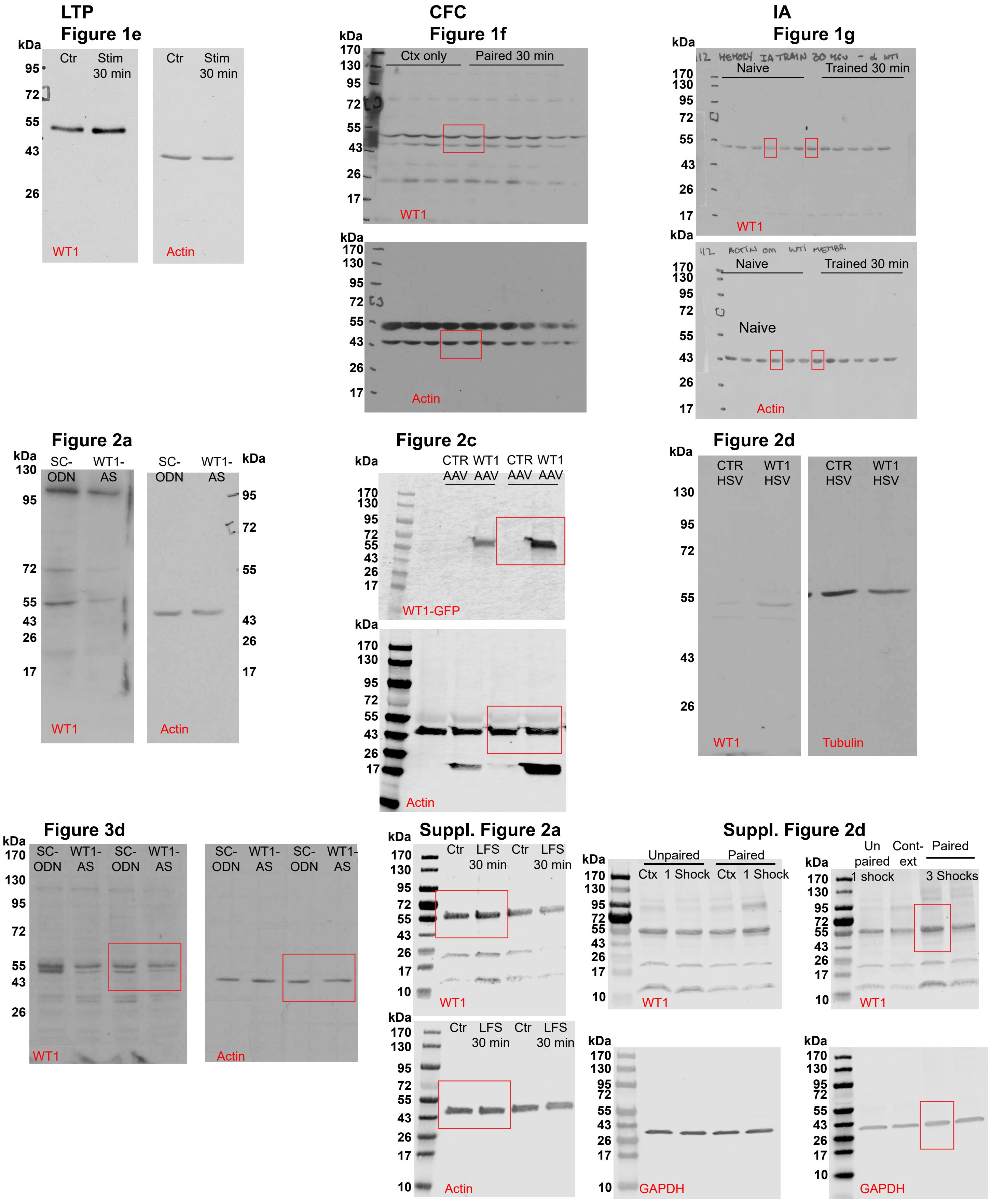

Supplementary Figure 10: Uncropped western blot images.
