# Supplementary material for: Wilm’s tumor 1 promotes memory flexibility
Source: Nat Commun. 2019 Aug 21;10:3756. doi: 10.1038/s41467-019-11781-x (PMC6704057; doi:10.1038/s41467-019-11781-x)
Supplement: Supplementary file 2 — Reporting Summary [file 41467_2019_11781_MOESM2_ESM.pdf]

nature research

Corresponding author(s): Chiara Mariottini, Cristina Maria Alberini, RAvi Iyengar

Last updated by author(s): Jul 30, 2019

Reporting Summary

Nature Research wishes to improve the reproducibility of the work that we publish. This form provides structure for consistency and transparency in reporting. For further information on Nature Research policies, see Authors & Referees and the Editorial Policy Checklist.

Statistics

For all statistical analyses, confirm that the following items are present in the figure legend, table legend, main text, or Methods section.

n/a	Confirmed	
☐	☒	The exact sample size (*n*) for each experimental group/condition, given as a discrete number and unit of measurement
☐	☒	A statement on whether measurements were taken from distinct samples or whether the same sample was measured repeatedly
☐	☒	The statistical test(s) used AND whether they are one- or two-sided<br>*Only common tests should be described solely by name; describe more complex techniques in the Methods section.*
☐	☒	A description of all covariates tested
☐	☒	A description of any assumptions or corrections, such as tests of normality and adjustment for multiple comparisons
☐	☒	A full description of the statistical parameters including central tendency (e.g. means) or other basic estimates (e.g. regression coefficient) AND variation (e.g. standard deviation) or associated estimates of uncertainty (e.g. confidence intervals)
☐	☒	For null hypothesis testing, the test statistic (e.g. *F*, *t*, *r*) with confidence intervals, effect sizes, degrees of freedom and *P* value noted<br>*Give P values as exact values whenever suitable.*
☒	☐	For Bayesian analysis, information on the choice of priors and Markov chain Monte Carlo settings
☒	☐	For hierarchical and complex designs, identification of the appropriate level for tests and full reporting of outcomes
☒	☐	Estimates of effect sizes (e.g. Cohen's *d*, Pearson's *r*), indicating how they were calculated

Our web collection on statistics for biologists contains articles on many of the points above.

Software and code

Policy information about availability of computer code

Data collection	Clampfit 10.6 (Molecular devices), EthoVision XT v13.0, MedAssociates
Data analysis	Prism Grappad v7, Matlab, ImageJ v1.47, PClamp v10, EthoVision XT v13.0

For manuscripts utilizing custom algorithms or software that are central to the research but not yet described in published literature, software must be made available to editors/reviewers. We strongly encourage code deposition in a community repository (e.g. GitHub). See the Nature Research guidelines for submitting code & software for further information.

Data

Policy information about availability of data

All manuscripts must include a data availability statement. This statement should provide the following information, where applicable:

- Accession codes, unique identifiers, or web links for publicly available datasets
- A list of figures that have associated raw data
- A description of any restrictions on data availability

The data that support the findings of this study are available from the corresponding authors upon request. Raw and processed mRNAseq data was uploaded to GEO Omnibus. Accessions code are:
- mRNA seq_ DEGs in LTP 90 min: GEO Series GSE120712
- mRNA seq_DEGs in Wt1Δ mice: GEO Series GSE120708

Code Availability:

Custom written Matlab scripts were used for the control theory-based model of WT1 function. The code is available upon request.

Field-specific reporting

Please select the one below that is the best fit for your research. If you are not sure, read the appropriate sections before making your selection.

☒ Life sciences ☐ Behavioural & social sciences ☐ Ecological, evolutionary & environmental sciences

For a reference copy of the document with all sections, see nature.com/documents/nr-reporting-summary-flat.pdf

Life sciences study design

All studies must disclose on these points even when the disclosure is negative.

Sample size	No statistical methods were used to predetermine sample size and the sample size reported in this manuscipt are comparable to similar literature in the field.
Data exclusions	Animals where cannulae were misplaced, viral expression spread outside of the hippocampus, and serious tissue damage was observed were excluded from the experimental groups.
Replication	Experiments were run at least three separate times. For the Wt1Δ-mRNA seq experiment, the results represent two different biological replicates. For behavior experiments the results are obtained from pulling together multiple animals from at least two different cohorts. Details of replicates are provided in each experiment.
Randomization	Animals were of the same gender and age and were randomly assigned to experimental groups.
Blinding	For all the electrophysiology and behavior experiments, the experimentalists were blind to the mice genotype or to the type of oligonucleotide or AAV/HSV virus treatment during the entire data gathering process. Only after the data were pooled and analyzed was the coding for the different groups revealed.

Reporting for specific materials, systems and methods

We require information from authors about some types of materials, experimental systems and methods used in many studies. Here, indicate whether each material, system or method listed is relevant to your study. If you are not sure if a list item applies to your research, read the appropriate section before selecting a response.

Materials & experimental systems

n/a	Involved in the study	
☐	☒	Antibodies
☒	☐	Eukaryotic cell lines
☒	☐	Palaeontology
☐	☒	Animals and other organisms
☒	☐	Human research participants
☒	☐	Clinical data

Methods

n/a	Involved in the study	
☒	☐	ChIP-seq
☒	☐	Flow cytometry
☒	☐	MRI-based neuroimaging

Antibodies

Antibodies used	Antibody source, dilution, catalogue number and unique identifier are listed in Supplementary Data 4
Validation	All antibodies used were validated for the applications described in this manuscript based on the guidelines from the manufactures datasheets.

Animals and other organisms

Policy information about studies involving animals; ARRIVE guidelines recommended for reporting animal research

Laboratory animals	Wt1Δ mice were bred in house (see Methods section for details).<br>Sprague-Dawley rats (6–8 weeks old) were purchased from Charles River Laboratory. Long-Evans rats (6–8 weeks old) were purchased from Harlan (Envigo).
Wild animals	Not applicable. None were used.
Field-collected samples	Not applicable. None were used.
Ethics oversight	All animals studies were approved and overseen by the IACUC (Institute for Animal Care and Use Committee) at Icahn School of

Ethics oversight	Medicine at Mount Sinai (ISMMS). Mount Sinai Animal Facilities are AAALAC certified for ethical and human treatment of animals.

Note that full information on the approval of the study protocol must also be provided in the manuscript.
