# Supplementary material for: Wilm’s tumor 1 promotes memory flexibility
Source: Nat Commun. 2019 Aug 21;10:3756. doi: 10.1038/s41467-019-11781-x (PMC6704057; doi:10.1038/s41467-019-11781-x)
Supplement: Supplementary file 3 — Description of Additional Supplementary Files [file 41467_2019_11781_MOESM3_ESM.pdf]

Description of Additional Supplementary Files

File Name: Supplementary Data 1
Description: Transcriptional changes (expressed as differentially expressed genes, DEGs) observed after 90 minutes from LTP induction.

File Name: Supplementary Data 2
Description: Enrichment analysis of transcriptomic changes predicts top regulatory transcription factors (TFs).

File Name: Supplementary Data 3
Description: Transcriptional changes (expressed as differentially expressed genes, DEGs) observed in the Wt1Δ mice.

File Name: Supplementary Data 4
Description: Complete list of antibodies used.
